# Artificial Intelligence Driven Innovation: Advancing Mesenchymal Stem Cell Therapies and Intelligent Biomaterials for Regenerative Medicine

**DOI:** 10.3390/bioengineering12121302

**Published:** 2025-11-26

**Authors:** Mengyu Huang, Waruna Lakmal Dissanayaka, Cynthia K. Y. Yiu

**Affiliations:** 1 Division of Paediatric Dentistry and Orthodontics, Faculty of Dentistry, The University of Hong Kong, 34 Hospital Road, Hong Kong SAR, China; huangmyu@connect.hku.hk; 2 Division of Applied Oral Sciences & Community Dental Care, Faculty of Dentistry, The University of Hong Kong, Hong Kong SAR, China

**Keywords:** mesenchymal stem cells (MSCs), artificial intelligence (AI), regenerative medicine, clinical translation, biomaterials

## Abstract

Artificial intelligence (AI) is revolutionizing regenerative medicine, particularly in advancing mesenchymal stem cell (MSC) therapies and smart biomaterials. This review highlights AI’s role in two core areas: First, at the biological level, AI can be used to predict MSC differentiation, immunomodulatory function, and therapeutic potential by analyzing multi-omics and imaging data, deciphering heterogeneity and improving precision. For instance, deep learning models based on MSCs’ morphology can successfully predict the differentiation propensity and uncover the regulatory networks underlying the intrinsic heterogeneity. Second, in engineering, AI shifts material design from trial-and-error to data-driven approaches. Machine learning models correlate material parameters with biological properties, enabling optimized screening. Furthermore, generative AI can be used to tailor novel materials through inverse design to achieve targeted properties like accelerated wound healing. However, the current development in this field remains constrained by several severe challenges, including the fragmented nature of existing research evidence, the insufficient reproducibility of model predictions in independent cohorts, and the significant translational gap from computational predictions to in vivo validation. Future research must not only demonstrate potential but also urgently address these fundamental and translational bottlenecks.

## 1. Introduction

The primary goal of regenerative medicine is to employ biological and engineering strategies to repair, replace, or regenerate tissues and organs that have been impaired due to aging, disease, or injury, with the ultimate aim of fully restoring their normal physiological functions [1]. Mesenchymal stem cells (MSCs) hold significant promise in this field owing to their multilineage differentiation potential, powerful immunomodulatory capabilities, and abundant paracrine signaling activities [2]. MSCs can migrate to sites of damage, differentiate into specific cell lineages, and secrete a variety of bioactive factors via paracrine mechanisms, thus facilitating tissue repair and modulating immune-inflammatory processes. These attributes render MSCs attractive therapeutic agents for a range of pathological conditions, including degenerative diseases such as Parkinson’s disease [3] and osteoarthritis [4], tissue injuries such as myocardial infarction [5], spinal cord injury [6], and pulp necrosis [7,8], as well as immune-mediated disorders such as rheumatoid arthritis [9] and graft-versus-host disease [10].

However, the clinical translation of MSCs faces several substantial challenges. Firstly, considerable functional heterogeneity exists among MSC populations due to variations in tissue sources such as umbilical cord, adipose tissue, and bone marrow, as well as differences in donor age and culture conditions, which directly compromises the reproducibility of therapeutic outcomes [11,12]. Secondly, the therapeutic effects of MSCs remain inconsistent across studies, and the underlying mechanisms have not been fully elucidated. A particularly critical limitation is the low post-transplantation survival rate; as the vast majority of administered cells undergo rapid apoptosis due to localized ischemic and hypoxic conditions, combined with host immune clearance, thereby hindering the attainment of sustained therapeutic efficacy [13,14]. Furthermore, the functionality of MSCs is highly dependent on their microenvironment [15], so biomaterials serve critical roles in stem cell-based therapies.

Functioning as artificial extracellular matrices, biomaterials not only provide essential three-dimensional scaffolding and mechanical support to promote cell survival and integration, but also actively regulate MSCs’ fate and function through microenvironmental cues [16]. As our understanding of MSCs–microenvironment interactions deepens, biomaterial design has progressed from providing passive structural support to enabling active biological guidance. In particular, intelligent biomaterials have emerged as a new paradigm, not only serving as cellular carriers but also acting as precise modulators of the cellular milieu [17]. These advanced materials function as responsive agents capable of sensing specific internal or external stimuli to activate therapeutic functions or enhance tissue regeneration [18,19].

Intelligent biomaterials are distinct from conventional biomaterials in several key aspects. First, they are stimulus-responsive and can react to triggers from both external and internal environments [19,20]. Second, they are functionally active, capable of releasing drugs, generating thermal effects, regulating cellular behaviors, and other therapeutic actions [21]. Moreover, they exhibit high design flexibility and tunability [22]. Through biomimetic engineering, these materials can mimic the mechanical and biochemical properties of the extracellular matrix. They can be engineered to respond to specific physiological or external signals such as pH, temperature, or light, and dynamically deliver cargo such as growth factors, cytokines, or nucleic acids. This dynamic regulatory capacity enables precise control over MSC homing, survival, proliferation, differentiation, and paracrine activity, thereby significantly improving the controllability and efficacy of regenerative therapies.

While significant progress in biomaterial research has improved the regenerative potential of MSCs in regenerative medicine, a comprehensive understanding of how surface chemistry, topological structure, and mechanical properties collectively influence MSC fate remains lacking. Key aspects such as the distribution of functional groups, pore architecture and surface roughness, as well as elastic modulus and matrix stiffness, are all believed to play interconnected yet not fully integrated roles [23]. These knowledge gaps not only impede the clinical translation of MSCs-based therapies but also motivate ongoing research to develop innovative strategies that address these limitations, with the ultimate goal of delivering tangible clinical benefits from stem cell advances.

The challenges faced by MSC therapies and smart materials, including cellular heterogeneity, unstable efficacy, and complex manufacturing processes, are fundamentally multidimensional and nonlinear system optimization problems that traditional methods struggle to effectively address. AI technology is emerging as a key solution, demonstrating unique advantages in processing high-dimensional omics data such as transcriptomics and proteomics, microscopic imaging data, including cell morphology and material structure, as well as complex dynamic processes [24,25]. AI can uncover deep patterns from massive datasets and establish predictive models that link donor cell fate, material parameters, and final therapeutic outcomes, thereby guiding optimal cell source selection and material design [26,27,28]. More importantly, AI enables reverse-engineered design of optimal formulations and structures for directive biomaterials while optimizing manufacturing processes to ensure batch consistency [29]. In summary, the advent of AI is poised to significantly accelerate the development and clinical translation of mesenchymal stem cell–smart biomaterial product systems.

This review provides an overview of innovative AI applications in MSCs research, spanning key areas such as multi-omics analysis, cell fate prediction, process optimization, and therapeutic efficacy assessment. It highlights how AI facilitates the rational design of intelligent biomaterials and discusses future directions and challenges in developing next-generation regenerative therapies that are both personalized and functionalized through deeper integration of AI and biological science.

## 2. Search Strategy and Outcome

The review followed the PRISMA guidelines. The primary search for article screening used in this review was conducted using Web of science (*n* = 557), PubMed (*n* = 542), Scopus (*n* = 711) and manual search (*n* = 154). Take PubMed as an example, we present our search strategy: (“Artificial Intelligence” [Mesh] OR “Machine Learning” [Mesh] OR “Deep Learning” [Mesh] OR “Neural Networks, Computer” [Mesh] OR “AI” OR “convolutional neural network*” OR CNN OR “generative adversarial network*” OR GAN OR “random forest” OR “support vector machine*” OR SVM OR “Bayesian optimization” OR “natural language processing” OR NLP) AND (“Mesenchymal Stem Cells” [Mesh] OR “Mesenchymal Stromal Cells” [Mesh] OR “Stromal Cells” [Mesh] OR MSC OR MSCs) OR (“Biocompatible Materials” [Mesh] OR “Biomimetic Materials” [Mesh] OR biomaterial* OR scaffold* OR hydrogel* OR “tissue engineering” [Mesh] OR “extracellular matrix mimics”) AND (“Regenerative Medicine” [Mesh] OR “regenerative medicin*” OR “tissue regeneration” [Mesh] OR “bone regeneration” [Mesh] OR “cartilage regeneration” OR “wound healing” [Mesh]). The detailed search strategy is provided in the Supplementary Materials.

**Figure 1:**
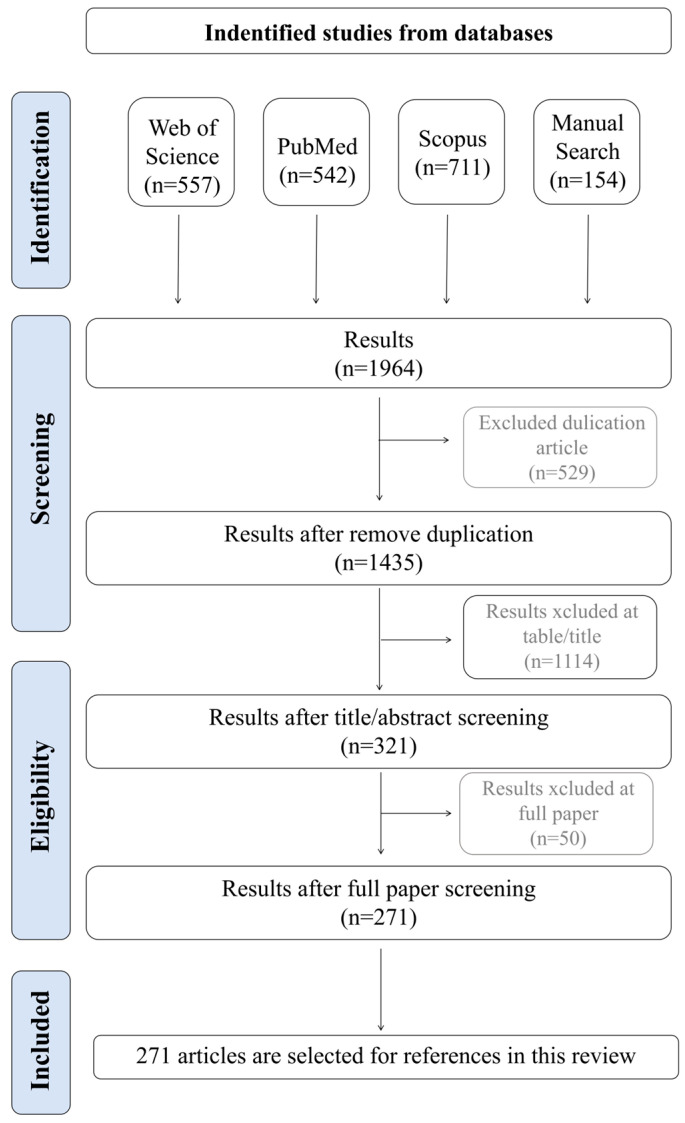
Search strategy. The flowchart outlines the search and screening process for the study. The search was first performed in Web of Science, PubMed, Scopus and manual search, then duplicate articles and articles that did not meet the research criteria were removed, and finally relevant articles were selected for references.

The literature screening was conducted collaboratively by two researchers. Following a rigorous selection process, 271 articles were selected for references in this study. The PRISMA flowchart (Figure 1) illustrates the identification and screening process of articles.

Based on the final results of this literature search, it can be observed that AI is increasingly being integrated with research on MSCs and biomaterials, catalyzing a shift from traditional, intuition-driven experimental approaches to a data-driven paradigm, thereby accelerating the rapid development of the field of regenerative medicine.

AI models are successfully decoding the complexity of MSCs by leveraging high-dimensional data from transcriptomics, proteomics, and live-cell imaging [30]. These models predict differentiation fate, resolve heterogeneity, and identify high-potency cell subpopulations [12], tasks that are difficult to accomplish efficiently with traditional analytical methods. Furthermore, AI is revolutionizing biomaterial design by establishing correlations between complex material parameters and biological performance [31], thereby transcending conventional trial-and-error approaches [32]. AI models are now being employed to inversely design novel smart biomaterials [25], which can be customized to elicit specific cellular responses such as enhanced osteogenesis or angiogenesis.

However, despite the growing demonstration of AI’s application potential in the field of regenerative medicine, the clinical translation of its achievements still has a long way to go. A significant gap persists between AI predictions and in vivo validation, with the vast majority of studies remaining confined to proof-of-concept demonstrations under controlled laboratory conditions. The field also grapples with the “black box” problem [33], where models offer high predictive accuracy but limited mechanistic interpretation, thereby restricting their utility for fundamental biological discovery. Furthermore, challenges such as data standardization, the reproducibility of models across different MSC donors and material batches, and the ultimate clinical translatability of these computational outcomes remain largely unresolved. The subsequent sections of this article will delve into the specific applications underpinning this promising yet precarious landscape, critically examining how AI is reshaping our understanding of MSCs and biomaterials, while emphasizing the considerable obstacles that must be overcome to realize its full transformative potential.

## 3. Main AI Algorithms for MSCs and Biomaterial Research

The application of AI in MSCs and biomaterial research relies on a solid foundation of diverse machine learning algorithms. A clear understanding of these computational tools is essential to appreciate how they convert complex data into biologically meaningful insights. This process usually starts with data acquisition and preprocessing, which is particularly crucial given the complex and multi-type nature of biological data. Data types range from structured experimental parameters and high-dimensional omics data, such as transcriptomics and proteomics, to unstructured image data, including microscopic images and material micro-CT scans. The quality and standardization of the data directly influence model performance.

**Figure 2:**
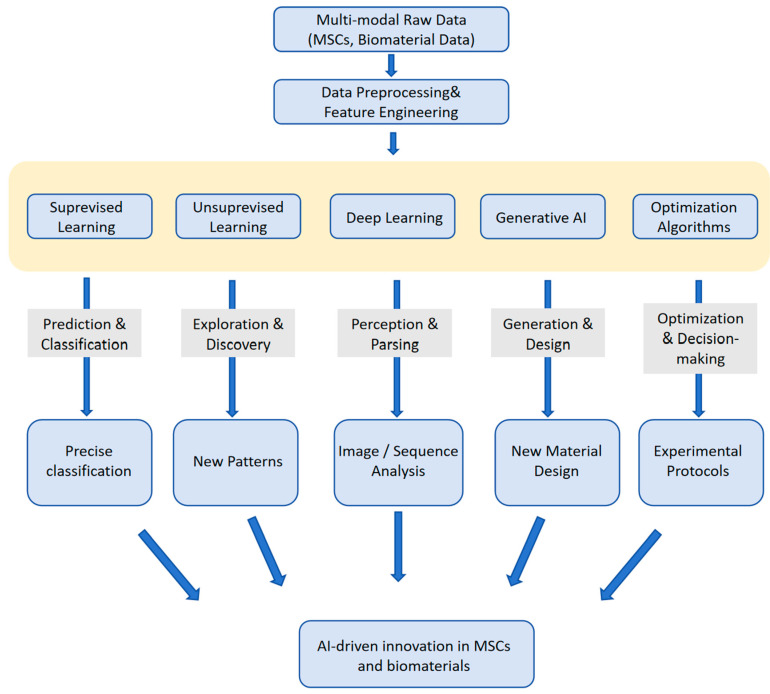
This figure illustrates the pivotal role of various categories of AI algorithms in translating heterogeneous raw data into scientific insights and practical applications. The process begins with the collection of multi-modal data. After essential data preprocessing and feature engineering, the data is processed by a core ensemble of AI algorithms, each suited to different tasks. The outputs of these algorithms facilitate data-driven decision making and discovery, ultimately guiding future experiments and clinical translation.

This section will introduce key machine learning algorithms that are advancing AI applications in MSCs and biomaterial research via Table 1 and Figure 2. We provide a concise overview of fundamental ML paradigms, with emphasis on core algorithm categories including supervised learning, unsupervised learning, deep learning, and generative AI. The discussion covers their basic principles and representative applications in the field, thereby establishing a necessary computational foundation for later chapters that delve into specific implementations.

## 4. Understanding MSCs Biology with AI: From Big Data to Mechanisms

MSCs demonstrate considerable therapeutic potential owing to their capacity for multi-lineage differentiation, immunomodulatory effects, and potent paracrine signaling. However, substantial donor-dependent heterogeneity, phenotypic instability during in vitro expansion, and functional variability pose major challenges to reproducible clinical outcomes and reliability. AI, especially machine learning and deep learning, is advancing the understanding of MSCs biology through multidimensional big data analysis with unprecedented precision and depth. This progress offers pivotal insights necessary for standardizing MSCs-based therapies and facilitating their precise clinical application.

### 4.1. Predicting Cell Fate and Therapeutic Potential

**Figure 3:**
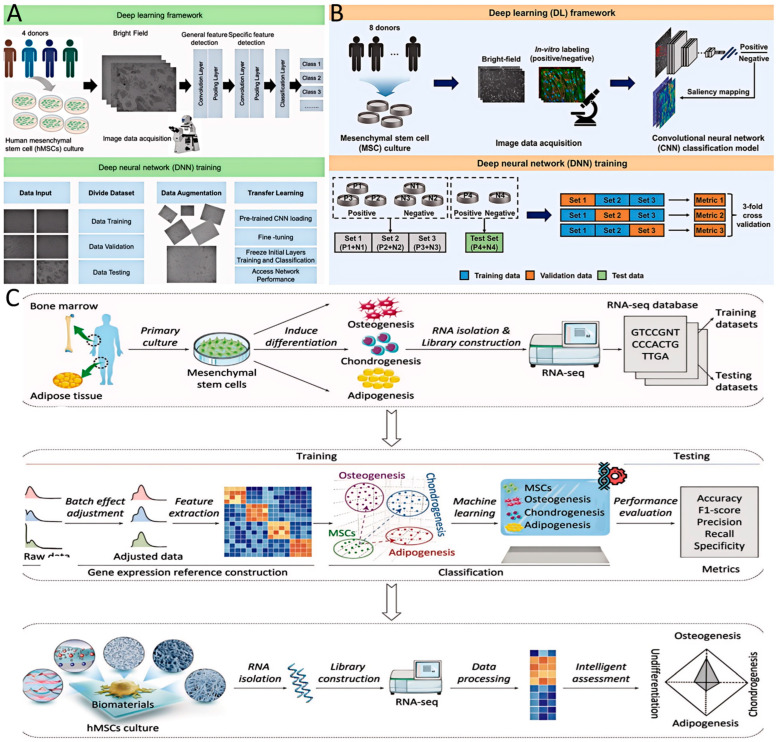
(**A**) Schematic illustration of the deep learning framework and deep neural network training process used to identify MSCs differentiation based on morphology. Reproduced with permission [37]. Copyright 2023, Frontiers Media S.A. (**B**) Schematic of the deep learning framework used to screen for functional MSC lines. MSC cultures were obtained from different donors for image data acquisition. To compare the classification performances of various CNN models, threefold cross-validation was conducted by splitting the dataset into non-overlapping subsets. Reproduced with permission [30]. Copyright 2022, Springer. (**C**) Overview of the workflow of the omics-based MSCs trilineage differentiation prediction model. First, collect the RNA-seq datasets related to tri-lineage differentiation of hMSCs from public databases and assign them into training and testing datasets. Then bioinformatic analysis was performed on training datasets comprising quality control, batch effect adjustment and feature selection to obtain the regenerative gene expression reference for hMSCs. Based on this, the assessment model was implemented to predict hMSC lineage fate based on machine learning. Then the performance of the assessment model was evaluated on testing datasets. Reproduced with permission [27]. Copyright 2023, Wiley.

Conventionally, evaluating the differentiation potential or immunosuppressive functions of MSCs requires time-consuming in vitro induction experiments that often take several weeks or involve complex molecular biology assays. In contrast, AI technology can utilize easily accessible early-stage data to achieve rapid and non-destructive prediction of cellular fate [27]. Computer vision-based AI methods have become powerful tools for predicting the developmental trajectories of MSCs. Convolutional neural network (CNN) is a deep learning architecture specifically designed to process grid-structured data such as images, which automatically extracts spatial features through convolutional layers and is widely applied in tasks including image recognition, classification, and object detection [34]. CNN can extract subtle morphological features from standard bright-field or fluorescence microscopy images, such as texture, shape, size, and intensity distribution, that are imperceptible to the human eye, and correlate these features with specific cell states [35,36]. After training, CNN models can accurately predict the subsequent osteogenic, adipogenic, or chondrogenic differentiation tendencies of MSCs by analyzing cell images taken just 24 h after seeding, achieving an accuracy of up to 90% (Figure 3A) [37].

For example, Kim et al. [30] employed a CNN to analyze live-cell microscopy images for predicting the functional status of MSCs. By training a CNN-based model, the study successfully classified MSC images into high or low multipotency stress-tolerant categories, enabling efficient cell screening (Figure 3B), achieving an accuracy of up to 92.2%. This non-invasive, image-based approach allows real-time and continuous monitoring of cell quality and fate prediction during culture, providing a transformative tool for selecting high-quality cells or early assessment of differentiation efficiency.

In addition, AI-driven methods based on omics data also offer reliable predictions of early MSC differentiation fate. Zhou et al. [27] developed a machine learning model named MeD-P, which integrated a large volume of publicly available RNA-seq data to construct a gene expression reference framework for tri-lineage differentiation in MSCs (Figure 3C). With the k-nearest neighbor algorithm, the model achieved high-accuracy prediction of differentiation lineages. Compared to conventional marker gene-based methods, MeD-P demonstrated superior accuracy, reaching 90.63% versus 80.21% on the test set, and could determine the differentiation tendency as early as the first week of hMSC culture. Similarly, Klontzas et al. [38] introduced an approach combining metabolomics and machine learning to predict the osteogenic differentiation potential of umbilical cord blood-derived MSCs in both two-dimensional and three-dimensional culture systems. XGBoost is a highly efficient gradient-boosting decision tree algorithm. It delivers outstanding performance in classification and regression tasks on high-dimensional and complex biomedical datasets [39], such as omics data, by iteratively training multiple weak learners and combining their predictions. By analyzing metabolic profiles and training an XGBoost model to distinguish between differentiated and undifferentiated states, the model achieved perfect accuracy in 2D conditions and maintained high performance in 3D cultures. These methods provide efficient and non-destructive tools for rapidly assessing the regulatory effects of biomaterials on stem cell fate, holding significant potential for applications in tissue engineering and regenerative medicine.

The clinical significance of the aforementioned AI-based, rapid, and non-destructive prediction models lies in the potential to serve as revolutionary tools for precise quality control and personalized medication in MSC therapy. Current clinical practice relies heavily on time-consuming in vitro induction assays, which require several weeks, to evaluate critical quality attributes such as the differentiation potential of cell products. These conventional methods are incompatible with the immediate infusion of fresh cells and cannot predict patient-specific therapeutic responses. In contrast, AI models could enable transformative clinical scenarios in the future. For instance, by rapidly acquiring and analyzing bright-field cell images prior to product release or administration, AI model could predict the osteogenic or chondrogenic potency of a cell batch within mere hours. This capability would facilitate the real-time selection of the most suitable cell products for conditions like osteoarthritis or bone defects, thereby minimizing ineffective treatments. Furthermore, by analyzing baseline transcriptomic or metabolomic data from donor MSCs, AI could predict the adaptability and therapeutic potential of different donor-derived cells in specific disease microenvironments. Looking ahead, clinicians might be able to match the most suitable MSC donor to a specific injury type or pathological state, a process analogous to blood type matching, thereby significantly improving treatment efficacy.

### 4.2. Revealing the Regulatory Network of Cell Fate Determination

**Figure 4:**
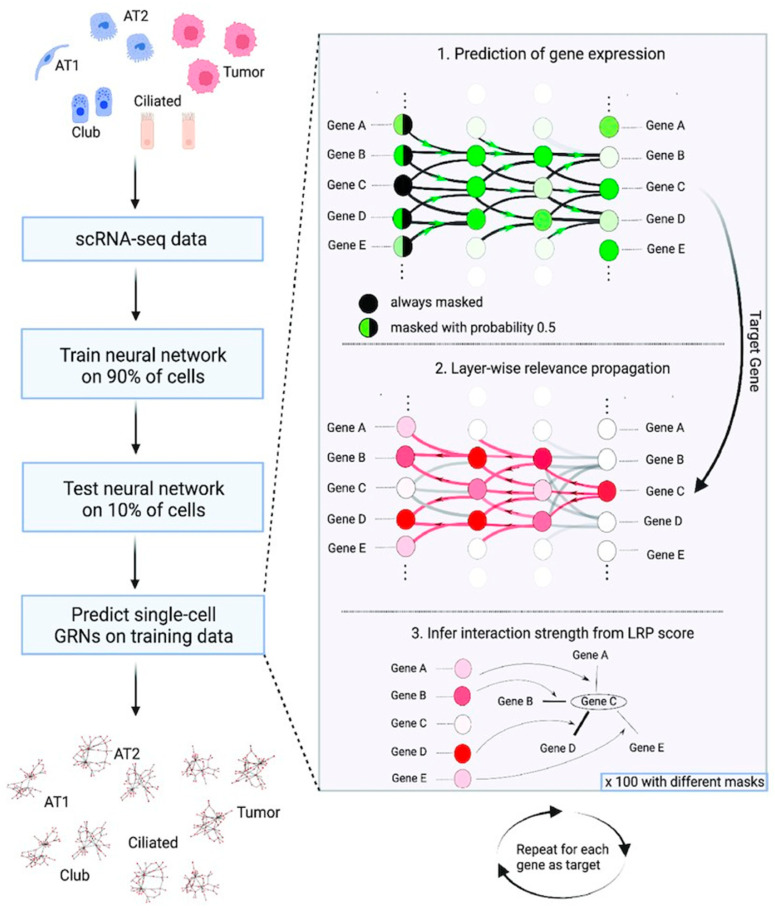
Workflow for the inference of single-cell GRNs by scGeneRAI. Reproduced with permission [47]. Copyright 2023, Oxford Academic. A neural network is trained on scRNA-seq data to predict each gene’s expression based on arbitrary sets of other genes. Following training, a single-cell GRN is predicted in three steps: (1) A target gene is predicted based on a set of other genes. (2) Logistic Regression Projection (LRP) is used to infer the relevance of every gene for this prediction. (3) The LRP scores subsequently serve as a measure of interaction strength between the target gene and all predicting genes. This procedure is repeated for 100 masks and for all genes as the target gene.

Beyond making predictions, the ultimate objective of AI in this field is to decipher the complex regulatory logic that governs MSCs’ fate determination. Gene regulatory networks (GRNs), which represent the intricate interactions between transcription factors and their target genes, are central to the control of cellular fate [40]. Conventional methods for inferring GRNs are often computationally intensive and fail to fully capture nonlinear regulatory relationships [41]. In contrast, AI-based approaches can reconstruct GRNs from time-series transcriptomic data with greater efficacy (Figure 4) [42,43]. These models help identify key transcription factors critical to differentiation or activation processes and simulate how such regulators cooperate to direct cells toward specific functional states [41,44,45]. For example, Kim et al. [46] developed a deep learning tool named DeepTFactor that predicts whether a protein acts as a transcription factor. By using convolutional neural networks to extract features from protein sequences, this tool can detect DNA-binding domains and other relevant characteristics, enabling high-accuracy transcription factor identification. Such data-driven reverse engineering strategies provide a dynamic and systems-level perspective for uncovering molecular switches and pathway interactions that guide MSC fate decisions, thereby establishing a theoretical foundation for designing precise cellular intervention strategies.

The AI-derived regulatory network governing MSC fate decisions holds profound clinical implications, as it provides a clear roadmap for developing precise “cellular reprogramming” and “targeted enhancement” therapies. While conventional MSC therapies involve transplanting whole cells with uncertain in vivo effects, the insights from this network are primarily speculative at this stage. Looking forward, AI-driven interpretation of the network may help identify key drug targets and facilitate the development of novel therapeutics. In next-generation genetically engineered MSC therapies, the network could serve as a blueprint for gene editing or transgenic modifications. With technologies such as CRISPR to precisely modulate key genes within the network may enable the creation of functionally enhanced MSCs for treating refractory tissue defects or autoimmune diseases. Furthermore, understanding this network could support the rational design of combined “material + cell + factor” strategies to maximize therapeutic outcomes.

### 4.3. Quantify and Analyze Cellular Heterogeneity

The emergence of single-cell RNA sequencing (scRNA-seq) and related technologies has revealed the remarkable transcriptional heterogeneity within MSC populations. The cellular heterogeneity challenge faced by MSCs refers to significant differences in gene expression, differentiation potential, immunomodulatory capacity, and secretory characteristics among MSCs [11]. This heterogeneity not only hinders precise functional analysis of MSCs in basic research but also severely constrains standardization and therapeutic stability in clinical applications.

The cellular heterogeneity challenge presents two core difficulties. First, the complexity of molecular mechanisms. This manifests as differences in epigenetic regulation and dynamic changes in metabolic states. For instance, a high-throughput single-cell DNA methylation and chromatin accessibility co-analysis study revealed that variations in DNA methylation and chromatin accessibility among cells within the same MSC population can lead to fluctuations in key gene expression [48]. Single-cell metabolomics research found that even within the same cell line, individual cells’ metabolic states may differ due to genetic, epigenetic, or environmental factors. This metabolic heterogeneity affects cellular functions and fate [49]. During stem cell differentiation, different subpopulations exhibit variations in metabolic activity, and changes in specific metabolites may determine cellular functions and fates [50,51,52]. Second, the diversity in microenvironment responses. Many transcription factor concentrations vary significantly between cells due to differences in synthesis, degradation, cell size, and shape, leading to distinct cellular responses [53]. Different subpopulations of mesenchymal stem cells may develop distinct functions and differentiation directions within the same microenvironment [54].

The heterogeneity of MSCs poses significant challenges while also underpinning their diverse functional capabilities. AI provides novel approaches to quantify, interpret, and harness this heterogeneity. t-SNE is a dimensionality reduction algorithm employed for the visualization of high-dimensional data, which uncovers clustering patterns by maintaining local pairwise similarities. Whereas UMAP represents a contemporary approach that effectively preserves local structure, captures global topological characteristics more accurately, and generally outperforms t-SNE in computational efficiency. The ability of both techniques to represent the intrinsic population architecture of high-dimensional single-cell data in a low-dimensional space renders them exceptionally useful for the identification of cellular subgroups possessing specific differentiation potentials or functional attributes [55]. By revealing hidden heterogeneities within MSC populations, these approaches enable optimized culture conditions tailored to specific subpopulations, improve expansion efficiency through targeted subpopulation selection, and enhance quality control by monitoring composition changes and functional consistency during manufacturing.

On the supervised side, machine learning models can construct predictive classifiers with transcriptomic data. These models not only precisely discriminate stem cells derived from different donors or tissue sources [56] but also forecast their responsiveness to specific induction factors [57]. This approach offers a promising strategy to address the issue of heterogeneity in cellular products.

For example, Liu et al. [58] developed an AI model combining hyperspectral imaging with a separable convolutional neural network (H-SCNN) to assess the functional status of human bone marrow-derived MSCs in a high-throughput manner. This model significantly surpassed manual visual inspection in performance metrics such as classification accuracy, AUC, F1 score, sensitivity, and specificity, while also reducing processing time from 60 min to 20 min. Weber et al. [59] proposed a deep learning-based image translation model that predicts fluorescence patterns of MSC senescence markers, such as SABG, p16, p21, and p38, directly from phase-contrast microscopy images. This non-invasive approach allows real-time senescence monitoring at single-cell resolution across multiple senescence induction methods, effectively capturing cell-to-cell heterogeneity.

These studies illustrate the emergence of AI as an efficient and scalable platform for quality control of MSCs, with promising applications in automating phenotypic analyses within cell therapy and regenerative medicine production pipelines. Collectively, these advances underscore the role of AI in enhancing the reproducibility of MSC-based therapies and streamlining biomanufacturing quality assurance.

**Figure 5:**
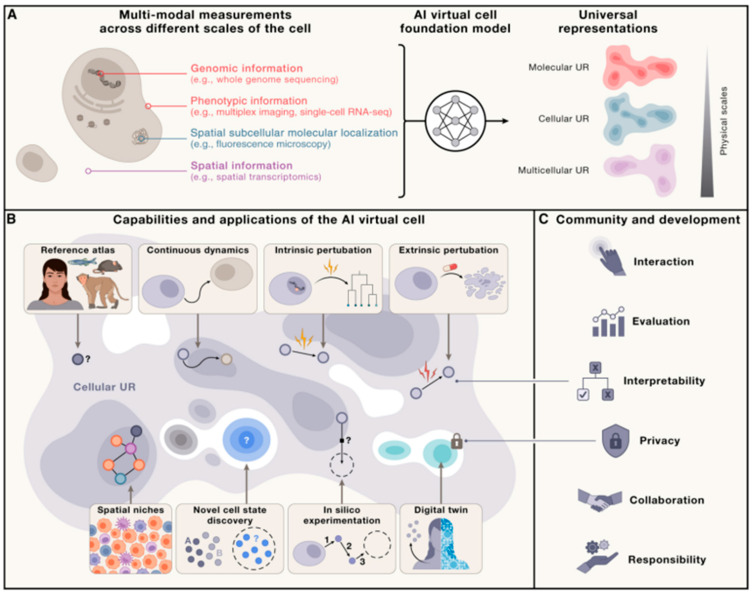
Artificial intelligence and omics enable the ambitious prospect of AI Virtual Cell (AIVC), a multi-scale multimodal neural network that models molecular, cellular and tissue behavior across states. Reproduced with permission [62]. Copyright 2024, Elsevier. (**A**) AIVC provides a universal representation of cell states, obtainable across species and conditions, and generated from diverse data modalities spanning multiple scales (molecular, cellular, and multicellular). (**B**) AIVC possesses the ability to represent and predict cellular biology. (**C**) The utility of AIVC depends on its interaction with humans across different levels.

Furthermore, generative adversarial networks (GANs) can produce synthetic single-cell data that are statistically consistent with real cellular datasets [60]. GANs learn through an adversarial game between a generator and a discriminator, which ultimately enables the generator to produce highly realistic data. This capability makes them particularly suitable for single-cell data analysis, as they can generate synthetic data to augment small, precious datasets, facilitate simulation studies, and protect patient privacy when sharing data. For example, Xu et al. [61] developed a method named scIGANs, which uses GANs to interpolate scRNA-seq data and mitigate dropout effects caused by technical artifacts. This method converts single-cell gene expression profiles into image-like representations, which GANs then process to generate biologically plausible expression values, effectively reconstructing missing gene measurements. Beyond augmenting valuable datasets for training more robust models, GANs also facilitate the creation of “virtual cells” that can be used to simulate perturbation experiments. These models allow researchers to computationally explore how specific genetic or environmental alterations influence overall cell states, thereby substantially reducing experimental costs and accelerating the discovery of underlying mechanisms (Figure 5) [62,63].

The core clinical significance of AI’s ability to predict MSC differentiation fate and decipher the heterogeneity lies in establishing a foundation for patient-specific personalized cell therapy products. This capability suggests a future in which MSC batches with high osteogenic potential can be selectively administered to patients requiring bone regeneration, or cell subpopulations exhibiting potent immunomodulatory activity can be chosen for immunoregulatory therapies such as graft-versus-host disease. Consequently, the predictability and success rate of treatments would be significantly enhanced, while the risk of ineffective interventions would be substantially reduced.

While image- or omics-based AI prediction models are promising, most remain at the proof-of-concept stage, with questionable generalizability. Their performance depends heavily on specific laboratory protocols, imaging equipment, and data preprocessing methods, posing significant reproducibility challenges across centers and donors. Moreover, these models are typically trained on “ideal” in vitro data and their accuracy in the complex in vivo microenvironment, characterized by inflammatory and hypoxic stresses, remains uncertain. Future work should prioritize prospective validation in large, multi-center cohorts and incorporate in vivo data to enhance model robustness and clinical applicability.

In conclusion, by integrating multimodal data, including imaging and omics, AI is transforming MSCs from an empirically applied cell therapy into an increasingly transparent and predictable system with well-defined biological mechanisms, quantifiable states, and controllable functions. This shift from predictive modeling to causal interpretation marks a crucial advancement toward precision medicine in MSC-based therapies, offering essential biological insights for refining stem cell manufacturing and guiding the design of intelligent biomaterials. However, key challenges such as limited generalizability, reproducibility, and the in vitro–in vivo gap hinder this potential. Despite these challenges, AI integration is fundamentally advancing MSC therapy toward a more predictable and mechanistic future.

## 5. AI-Driven Design of Intelligent Biomaterials

The development of traditional biomaterials has long relied on researchers’ expertise and iterative experimental approaches, often described as trial and error. This process tends to be time-consuming, costly, and limited in its ability to systematically optimize complex parameters. A particular obstacle is the lack of comprehensive analysis of material-cell interactions, which significantly restricts progress in biomaterial research [64,65]. For instance, in the case of bone-repair hydrogels, the number of theoretically possible parameter combinations, such as choices of raw materials, degree of cross-linking, pore size, and degradation rate, is exceedingly large [66]. Conventional experimental methods can only explore a small subset of these combinations, requiring considerable investment of time and resources.

**Figure 6:**
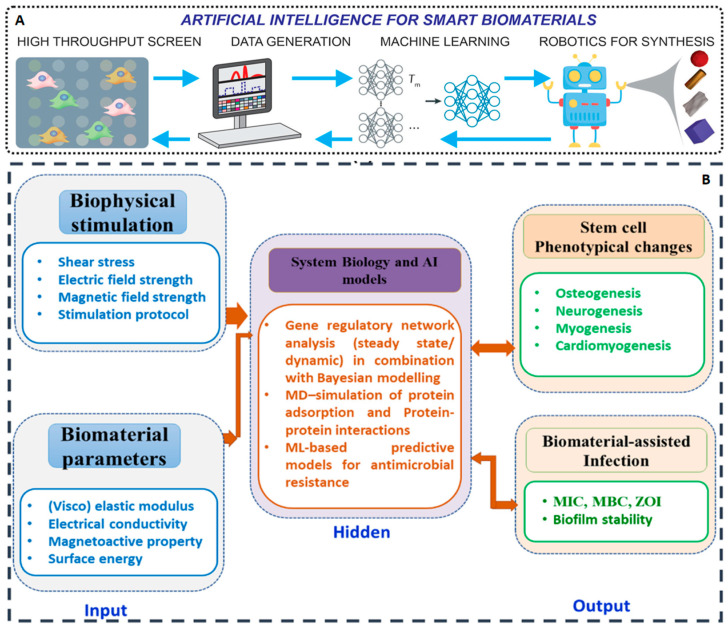
(**A**) Workflow of AI-assisted smart biomaterial design, synthesis, and analysis. Reproduced with permission [25]. Copyright 2025, Wiley. (**B**) Schematic to illustrate the role of system biology and AI-based modeling approaches to establish high-throughput stem cell phenotypical switching to different lineages (bone/neural/cardiac/muscle) or antimicrobial resistance, based on biomaterial and biophysical stimulation parameters. Reproduced with permission [68]. Copyright 2022, Elsevier.

Moreover, the relationship between micro-scale characteristics, such as polymer chain entanglement, and macro-scale properties, like elastic modulus, remains poorly quantified, often leading to repetitive and inefficient experimental cycles. Literature analyses indicate that, between 2002 and 2021, the majority of bone repair material studies focused on incremental improvements within conventional material systems, rather than pioneering novel tissue engineering strategies. This highlights the innovation constraints inherent in traditional methodologies [67]. The incorporation of AI is now reshaping this paradigm, shifting biomaterial design from an experience-dependent practice to a data-driven and rational process (Figure 6A). By deciphering complex structure–property relationships and biological responses, AI facilitates the development of a new generation of intelligent biomaterials capable of actively directing cellular behavior, responding to environmental cues, and ultimately improving tissue regeneration outcomes.

### 5.1. Rational Design of Material Properties

The key characteristics of biomaterials, such as chemical composition, microstructure, stiffness, surface topography, charge, and hydrophobicity, collectively shape the microenvironment that governs cellular behaviors including adhesion, spreading, proliferation, and differentiation [69]. However, the interactions between these parameters and cell fate occur in a highly complex and nonlinear manner that exceeds the capacity of human intuitive reasoning.

Machine learning models, especially supervised learning algorithms, are well suited to address this challenge. Researchers can compile databases containing a wide range of material formulations along with their corresponding in vitro and in vivo experimental outcomes. Using algorithms such as random forests, support vector machines, and neural networks, they can train models to quantify structure–activity relationships between material parameters, structures, and functional properties [70]. For example, computational approaches including molecular dynamics simulations, finite element analysis, and machine learning enable prediction of hydrogel properties, such as mechanical strength, biocompatibility, and stimulus responsiveness, across molecular and macroscopic scales [71]. A well-trained model can accurately predict the capacity of a biomaterial to support MSCs differentiation based on its physicochemical characteristics, elucidate how various factors affect differentiation outcomes, and suggest optimized formulations (Figure 6B) [72,73]. This capability allows the virtual screening of thousands of candidate material compositions prior to synthesis, substantially reducing experimental workload and rapidly narrowing down the most promising candidates for further validation [74]. Robust and generalizable models ensure that predictions remain valid under varying experimental conditions and for unseen data, increasing confidence in pre-screening outcomes. As a result, the integration of machine learning technology streamlines biomaterial development for stem cell applications and supports faster clinical translation in regenerative medicine and cell therapy.

The AI-driven rational design of biomaterials holds clinical potential by enabling customized solutions for specific tissue defects and patient groups in the future. For example, with medical imaging data, AI could generate personalized scaffold designs that match the mechanical and geometric properties of the defect, improving implant stability and biocompatibility. AI may also optimize materials for specific clinical needs. In osteoporotic patients, it could develop materials with lower modulus and higher porosity to promote cell ingrowth and vascularization, thus reducing stress shielding and accelerating healing. For cartilage repair, AI might adjust hydrogel water content and lubrication to help restore joint function. As these clinical scenarios become reality in the future, AI is poised to transform the therapeutic effectiveness of biomaterials.

### 5.2. Reverse Design and Discovery of New Materials

Generative AI is paving the way for a new paradigm of “reverse design” in biomaterials, going beyond the analysis of existing datasets. Generative AI is a branch of artificial intelligence whose core function is to learn the intrinsic distribution patterns of existing data and, based on this, generate novel data samples that are similar to but not identical to the original data. Its ability to enable “inverse design” stems from its capacity to learn from complex “property-structure-process” relationships, using desired material properties as input conditions. After training on massive datasets, the model can directly and inversely deduce novel molecular structures or material compositions that fulfill these specified property requirements [75].

For instance, Jiang et al. [76] developed an AI platform for designing antimicrobial peptide hydrogels aimed at treating drug-resistant bacterial infections such as *MRSA* and *Escherichia coli*. Through generative design and multi-objective optimization, the platform proposed novel thiol-containing antimicrobial peptides. These were integrated with hydrogels and copper-modified barium titanate to form composite materials that demonstrated strong antibacterial efficacy and promoted wound healing. Experimental results confirmed that the AI-designed hydrogel significantly enhanced antimicrobial performance and accelerated wound closure in both in vitro and animal models, underscoring the potential of AI in guiding functional biomaterial development.

Despite the fact that the inverse design capability of AI has accelerated the discovery of new materials at an unprecedented pace, it has concurrently introduced a critical validation gap. The numerous virtual material candidates generated by AI ultimately require synthesis and functional validation through time-consuming and labor-intensive wet laboratory experiments. Currently, cases successfully completing the full research chain from virtual design to experimental synthesis and in vivo functional confirmation remain exceedingly scarce. Most studies are still confined to computational simulations or preliminary in vitro testing, with a severe lack of systematically evaluated data on efficacy and safety in disease animal models. This translational void between in silico predictions and in vivo outcomes represents one of the most fundamental challenges in AI-driven materials science.

Although current applications of AI-driven reverse design in recycled biomaterials remain relatively limited, successful examples in drug discovery and other biomaterial domains highlight its broad applicability [77]. We anticipate that AI will continue to transform the design of recycled biomaterials, enabling the on-demand customization of materials tailored to specific clinical needs. This advancement is expected to provide innovative momentum for the future of regenerative medicine.

### 5.3. Intelligent Feedback Assisted Design of Dynamic and Responsive Materials

Conventional biomaterials generally exhibit fixed mechanical properties, in contrast to the highly dynamic nature of the extracellular matrix (ECM). Static materials often fail to adequately mimic the continuous remodeling processes inherent to biological tissues [64]. For example, the mechanical behavior of many biomaterials does not fully match the requirements of the native tissue. Commonly used bone regeneration materials such as polycaprolactone (PCL) degrade slowly; some PCL scaffolds require three to four years to fully dissolve, which misaligns with the rate of replacement and resorption during new bone formation [78]. Furthermore, traditional bone regeneration scaffolds often possess a stiffness greater than that of natural bone, which can lead to insufficient mechanical stimulation and result in bone resorption due to stress shielding effects, ultimately compromising regenerative outcomes [79].

Biological tissues often display hierarchically porous architectures. Different tissue types and developmental stages demand distinct pore sizes, a degree of structural sophistication that remains difficult to replicate with synthetic materials. In the context of bone regeneration, natural cancellous bone contains complex and interconnected pores, whereas traditional repair materials such as hydroxyapatite ceramics often show limited porosity and poor interconnectivity, potentially hindering cell migration and nutrient transport [80].

Another critical shortcoming of conventional biomaterials is their limited ability to monitor and respond to microenvironmental changes in real time. For instance, natural bone tissue can self-repair through the coordinated activities of osteoclasts and osteoblasts, whereas calcium phosphate ceramic implants lack such autonomous repair capabilities [81]. Similarly, traditional drug delivery systems such as PLGA microspheres release therapeutic agents mainly through passive diffusion or polymer degradation [82]. They lack the ability to dynamically adapt to changes in the local microenvironment, such as variations in pH or enzymatic activity. In contrast, the ECM demonstrates an innate capacity to respond to these dynamic conditions.

Therefore, novel biomaterials need to evolve into dynamic systems with tunable properties that can adjust biophysical signals over time, which is essentially a “fourth-generation” of materials capable of sensing and adaptively responding to changes in the physiological microenvironment [23,68]. AI plays an indispensable role in designing and optimizing such complex systems. For example, Wang et al. [83] proposed a hybrid model that integrates physical simulation and AI to predict drug release behavior in biomaterials. Unlike traditional methods that rely on fixed parameters and static diffusion models, particularly models such as NODE and SHAP can dynamically learn nonlinear relationships in the drug release process and adjust prediction results in real time based on spatial location and material properties. This approach not only improves predictive accuracy but also enhances the controllability and personalization of drug release, offering a more flexible and efficient solution for the design of intelligent drug delivery systems.

By analyzing existing biomaterial datasets, AI can predict the properties of new biomaterials and even create entirely new compositions and structures tailored to specific functional requirements. For instance, AI can generate innovative biomaterial constructs, such as hydrogels or 3D scaffolds, with defined mechanical properties and degradation rates suited to the target application. Furthermore, AI facilitates the design of smart materials that respond to microenvironmental cues such as pH or mechanical stress, laying the groundwork for precision medicine [25]. AI also holds promise for integrating multi-scale data spanning molecular, cellular, and material levels to construct virtual models of cell–material interactions. This capability will help shift mechanistic studies from reductionist approaches toward a more systemic perspective [62,63].

In summary, AI serves not merely as an accelerator but as a fundamental driver of innovation in biomaterials research in the future. By deciphering complex datasets, constructing predictive models, and generating novel design solutions, AI enables the development of increasingly sophisticated generations of intelligent biomaterials. These materials function beyond passive cell carriers; they act as active partners that interact with implanted MSCs. By working in concert to sense, respond to, and remodel damaged microenvironments, MSCs and biomaterials collectively enhance the therapeutic efficacy of regenerative medicine. This progress will open new avenues for building complex tissue-engineered constructs and advancing the next generation of personalized implantable medical devices.

## 6. Challenges and Perspectives

Although AI has demonstrated considerable promise in advancing research on MSCs and intelligent biomaterials, its comprehensive integration into the biomedical field continues to encounter numerous significant translational challenges and ethical concerns that must be thoroughly addressed.

### 6.1. Data Quality and Standardization Issues

The development of AI models critically depends on high-quality, standardized, and well-annotated datasets. However, a fundamental limitation in the intersection of medicine and AI is the pronounced lack of large-scale, standardized, multi-center biomedical datasets [84]. Most current studies, including many cited in this review, are built upon data generated from single laboratories, specific donor sources, or controlled in vitro conditions. This singular data origin leads to significant data fragmentation, resulting in widespread issues such as high noise, pronounced batch effects, and source heterogeneity [85]. Consequently, models are highly susceptible to learning these non-biological technical variations, leading to overfitting and weak generalizability. A predictive model demonstrating exceptional performance in one study often fails to generalize for data from different sources, raising major concerns regarding the reproducibility and robustness of AI applications. Therefore, the transition from proof-of-concept research to clinically reliable tools necessitates validation in independent and heterogeneous cohorts. Establishing AI systems with strong generalizability must be founded upon large-scale, standardized, multi-center datasets, which remains a critical and unmet need in the field.

### 6.2. Model Interpretability and Mechanistic Insight Lackage

A further major limitation concerns the lack of algorithmic interpretability. Many deep learning models function as “black boxes,” with decision-making processes that lack transparency [33]. In biomedical applications, merely obtaining accurate predictions is insufficient; it is equally crucial to reveal the biological mechanisms underlying these predictions to establish scientific trust and drive new discoveries. Explainable Artificial Intelligence (XAI) presents a promising pathway to address this challenge [86]. Within MSC and intelligent biomaterials research, XAI methods, such as LIME and SHAP, can be applied to trained black-box models. These techniques utilize local approximation or perturbation analysis to identify the input features most critical to specific predictions, like instance key genes, material parameters, or culture conditions. This capability directly addresses the fundamental question of which factors the model relies on for its decision-making.

A representative example can be found in the study by Chung et al. [87], who developed a machine learning workflow to predict cell viability on conductive MXene biointerfaces. After training an artificial neural network model, the researchers applied SHAP analysis to quantify the direction and magnitude of influence of four key input parameters, including pc-MXene loading, peptide loading, applied voltage, and stimulation frequency, on the model-predicted cell viability. The SHAP interpretation revealed a strong negative impact of high voltage on cell survival, which was confirmed by fluorescence microscopy observations showing substantial cell detachment from the biointerface when the voltage was increased to 3.0 V. Furthermore, SHAP indicated that the negative effect of pc-MXene loading was attenuated under higher voltage conditions, prompting further investigation. This led to the discovery that the phenomenon might be associated with the aggregation of pc-MXene at specific concentrations, which altered interfacial conductivity and peptide adhesion stability. Thus, in this study, XAI successfully transformed a complex ANN black-box model into an interpretable tool that not only generated predictions but also elucidated the intricate nonlinear interactions between parameters and cellular activity, directly guiding subsequent mechanistic experiments and advancing the transition from data-driven prediction to biological mechanism discovery.

While this review identifies XAI as a promising future direction to address this issue, current applications of XAI in the field still remain superficial. Simply providing feature importance rankings and directional influences falls short of delivering a causal, mechanistic understanding of the underlying biology or material–cell interactions. There is a risk that AI may identify spurious correlations without revealing genuine regulatory principles, thereby limiting its utility for generating novel, testable biological hypotheses.

### 6.3. The Clinical Translation Bottleneck

Currently, most AI-driven MSC research and biomaterial design relies predominantly on data generated under idealized experimental conditions, such as two-dimensional cell cultures and homogeneous materials. Models trained on such data fail to adequately capture key physiological factors present in real in vivo environments, including immune responses, vascularization, and dynamic changes in the tissue microenvironment. This dependence on idealized data severely constrains the predictive power of AI models in real clinical settings and may lead to overly optimistic assessments of technological maturity.

Moreover, even when models demonstrate excellent predictive performance in vitro, most research outcomes remain confined to the in vitro experimental stage and lack systematic validation in animal models or human studies [85]. Particularly in the context of reverse design, the materials or cellular strategies generated by AI require functional and safety assessments through complex, time-consuming, and costly in vivo experiments. This validation gap consequently leaves numerous AI-driven regenerative medicine solutions largely theoretical, hindering their translation into practical clinical applications.

Furthermore, current models predominantly rely on statistical correlations and lack clearly defined decision thresholds that are directly linked to clinical outcomes. For instance, a model’s prediction of “high osteogenic potential” must be quantitatively correlated with actual patient bone healing rates to possess meaningful clinical utility. Such studies remain largely absent, forming a critical bottleneck that impedes the transition of AI from an academic tool to a clinically approved medical product.

### 6.4. Ethical and Regulatory Challenges

The application of AI in biomedicine raises a series of ethical and social concerns. Data privacy and security constitute a paramount concern [88], particularly when handling patient genomic and clinical information. Furthermore, the risk of algorithmic bias is significant. If training data fails to adequately represent diverse populations, models may exacerbate healthcare disparities [89,90].

**Figure 7:**
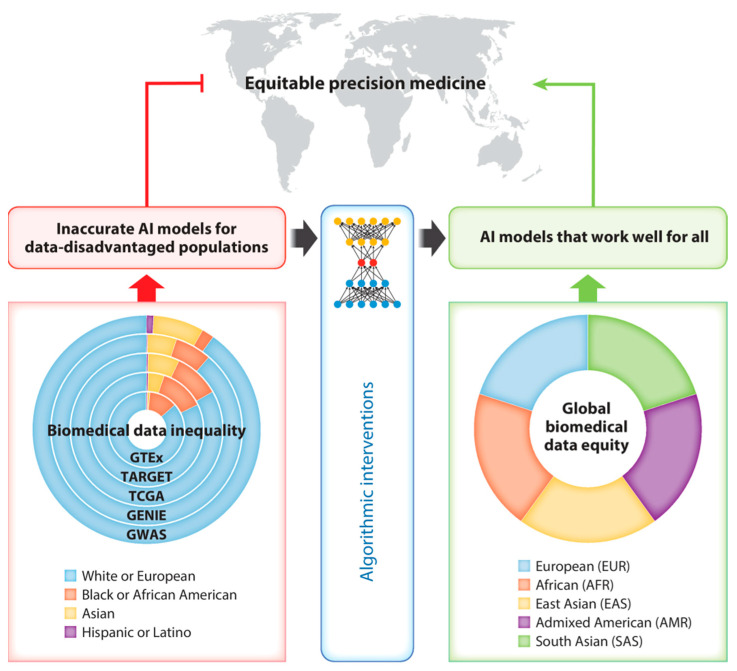
Addressing data inequality challenges in AI-driven precision medicine [91]. Copyright 2023, Annual Reviews.

For instance, research by Gao et al. [91] highlights that while biomedical data forms the foundation for developing medical AI models, over 80% of Genome-Wide Association Studies data originates from European populations, who represent less than 20% of the global population (Figure 7). This predominant reliance on data from European ancestries results in models with reduced accuracy and poorer performance for non-European populations in areas such as disease prediction and treatment recommendation. The mismatch between training data distribution and target populations also severely compromises model generalizability. Through comprehensive data analysis, Gao et al. demonstrate that while AI holds potential to accelerate precision medicine, it may simultaneously amplify health risks stemming from data inequality.

From a regulatory perspective, authorities in various countries have begun establishing approval frameworks for AI-based medical software [92]. A landmark example of real-world translation is the IDx-DR system [93]. Focused on the automated screening of diabetic retinopathy, it received marketing authorization through the U.S. Food and Drug Administration’s (FDA) De Novo pathway in January 2018. This approval marked IDx-DR as the first autonomous AI system cleared by the FDA capable of providing definitive clinical diagnostic decisions, such that it could output results without requiring secondary interpretation of the images by a clinician. A pivotal factor in its authorization was a rigorous prospective, multicenter clinical validation study conducted within real-world primary care settings, which successfully demonstrated the algorithm’s high sensitivity and specificity in identifying referable diabetic retinopathy. This case clearly illustrates a successful transition from proof-of-concept to a clinically reliable tool: it addresses the critical healthcare need of mitigating specialist shortages and improving screening accessibility, while also having passed stringent regulatory scrutiny, thereby paving the way for the development and approval of subsequent autonomous AI diagnostic systems. It serves as an invaluable paradigm for clinical translation in the field of AI for regenerative medicine. However, despite this considerable potential, challenges such as the frequent updates of AI models, the opaque decision-making processes, and difficulties in controlling data biases complicate safety verification and the establishment of clinical trust, making this an evolving and complex landscape. Consequently, the path toward the widespread clinical integration of AI models remains a considerable journey ahead.

### 6.5. Perspectives

Addressing these challenges will require sustained interdisciplinary collaboration among biologists, clinicians, computational scientists, ethicists, and regulatory agencies. Only through the collective establishment of robust, equitable, transparent, and trustworthy AI systems can we fully realize their potential and safely usher regenerative medicine into an intelligent new era. Future efforts should prioritize the development of a collaborative, reliable, and efficient intelligent research ecosystem. Key to this endeavor is the formation of cross-institutional multimodal data alliances that leverage privacy-preserving technologies such as federated learning to overcome data fragmentation and heterogeneity, thereby providing a solid foundation for model training [94]. At the same time, explainable AI must be deeply embedded within research frameworks to convert high-accuracy predictive models into interpretable insights concerning biological mechanisms, facilitating a transition from opaque black-box systems to transparent and verifiable approaches [95]. In terms of methodology, increased emphasis should be placed on hybrid models that integrate generative AI with established physical principles, enabling not only data enhancement but also the generation of novel and testable hypotheses for biomaterial design and cellular reprogramming [96,97].

To ensure a smooth translation from laboratory research to clinical applications, it is essential to work closely with regulators to develop adaptive review frameworks and standardized good machine learning practices tailored to the dynamic nature of AI technologies [98]. Ultimately, these advancements are expected to converge into automated and self-directed laboratories that combine AI-driven decision making, robotic automation, and high-throughput characterization within a closed-loop system. Such an integrated platform will effectively bridge intelligent design with physical realization, significantly enhancing research and development efficiency while systematically accelerating the intelligent transformation of regenerative medicine.

## 7. Conclusions

This review systematically examines the current applications and impact of AI in MSC research and the development of intelligent biomaterials. At the fundamental research level, AI has demonstrated powerful data processing capabilities, enabling the establishment of predictive models for MSC differentiation fate and functional potential through the analysis of microscopic images and omics data. At the engineering application level, AI-driven rational design models have preliminarily achieved a quantitative mapping from material parameters to biological performance, while generative AI has validated, on a small scale, the feasibility of its “inverse design” concept for novel materials, such as the peptide-based hydrogels tailored for specific properties like antibacterial and pro-healing functions.

However, it must be emphasized that most of these encouraging achievements remain largely confined to the early-stage research phase. The research evidence reviewed in this article also clearly highlights major challenges in the field: the reproducibility and generalizability of AI models are constrained by highly heterogeneous and fragmented data; the black-box nature significantly impedes the discovery of interpretable biological mechanisms; and most critically, a substantial translational gap exists between computational predictions and in vivo efficacy validation.

Therefore, future progress requires addressing these fundamental bottlenecks beyond algorithmic advances. This requires establishing interdisciplinary, standardized data platforms to enhance data quality and comparability; advancing explainable AI to transform high-accuracy predictions into verifiable biological insights; and ultimately conducting rigorous, systematic in vivo experiments to bridge the critical evidence gap between in silico designs and in vivo therapeutic outcomes. Only through such foundational work can AI evolve from a powerful tool into a reliable engine driving precision and intelligence in regenerative medicine.

## Figures and Tables

**Table 1:** Summary of the common AI algorithm in MSCs and biomaterials research.

Classification	Typical Algorithms	Algorithm Description	Role in Research	Examples of Specific Application Scenarios
Supervised learning	Random Forest/XGBoost	Leveraging the strength of decision tree ensembles, these robust algorithms are particularly adept at handling structured data and evaluating feature importance. This capability allows it to integrate heterogeneous experimental data and identify the most critical material properties or biological signatures that dictate cell fate and function.	To build predictive models that decipher the complex, nonlinear relationships between biomaterial parameters and MSC behavior, thereby accelerating rational design.	1. Predicting the differentiation fate of MSCs based on material parameters, such as hardness and chemical composition. 2. Predicting clinical efficacy of MSC treatment and discovering key biomarkers.
	Support Vector Machine (SVM)	This algorithm is designed to identify the optimal classification boundary in high-dimensional spaces, demonstrating strong capability in handling such complex data. This makes it particularly suited for classifying cell subtypes or material types based on complex omics or spectral data.	To perform precise classification tasks, such as distinguishing MSC donor sources or potency based on their molecular profiles, or categorizing biomaterial formulations.	This algorithm enables the precise classification of MSCs into osteogenic-prone or adipogenic-prone lineages based on their omics signatures, thereby prescreening them for specific therapies.
Unsupervised learning	PCA/t-SNE/UMAP	This algorithm generates low-dimensional visualizations of high-dimensional data, enabling researchers to intuitively discover inherent clustering patterns within the data, such as unlabeled cell subpopulations or groups of biomaterials with similar properties.	This algorithm uncovers the hidden structure within complex datasets. It creates a visual map that makes it easy to see natural clusters, for example, to find new cell subtypes or group similar biomaterials together.	1. Analyze scRNA-seq data, visualize and discover unknown functional subgroups of MSCs. 2. Perform dimensionality reduction on diverse material formulations to observe the natural clustering patterns and identify groups of formulations with similar properties.
	K-means clustering	An algorithm that automatically groups data points into distinct clusters, ensuring that points within the same cluster are as similar as possible. This algorithm performs unbiased clustering, automatically revealing hidden groups within your data. It can identify distinct cell subpopulations or categorize material types purely from the data itself, without any prior labeling.	It discovers new categories by performing automated, unlabeled sorting of cell populations or materials, which directly enables data-driven phenotyping of heterogeneous MSC cultures and the categorization of biomaterial datasets.	1. Mixtures of cells can be automatically clustered according to the expression profile of surface markers. 2. A large number of polymer materials can be clustered to quickly screen out the material categories with similar characteristics.
Deep learning	Convolutional Neural Network (CNN)	As a network architecture specifically designed for image processing, it excels at automatically extracting spatial hierarchical features. This capability makes it ideal for analyzing cell morphology and material microstructure in microscopy or SEM images.	To analyze the morphology of cells and the microstructure of materials, and to make image-based predictions and classifications.	1. The apoptosis status or differentiation tendency of MSCs can be predicted non-invasively according to the morphology of MSCs in micrographs. 2. Analyze the SEM image of biological material and predict its porosity or mechanical strength.
	Recurrent Neural Network (RNN/LSTM)	A network architecture specifically designed for sequential data, capable of capturing temporal dependencies through its built-in memory mechanism, perfect for analyzing dynamic biological processes that evolve over time.	Its core function is to model and forecast time-series data, capturing the dynamic processes that evolve over time. This allows for the prediction of future states in MSC culture, such as growth dynamics, or biomaterial performance, such as long-term degradation profiles.	1. Analyze the dynamic change in cell growth or differentiation to predict its future state. 2. To predict the degradation kinetics of biomaterials in vivo.
Generative AI	Generative Adversarial Network (GAN)/Variational Autoencoder (VAE)	A generative model that learns the underlying distribution of complex data to generate novel, realistic samples, such as synthetic cell images or new molecular structures with desired properties.	1. Data enhancement: To overcome data scarcity for rare cell states or material types by generating synthetic data to expand the dataset. 2. Reverse design: to create new materials or molecular structures with desired properties.	1. Generate realistic virtual cell microscopy images to increase the amount of training data. 2. Inversely designed a novel peptide hydrogel molecular structure that can promote angiogenesis to the greatest extent.
Optimization algorithm	Bayesian Optimization	A sequence optimization strategy based on Bayes’ theorem is proposed to find the optimal solution of complex functions with a minimum number of trials. As a sample-efficient strategy, it addresses the challenge of optimizing expensive-to-evaluate experiments. By intelligently selecting the most promising trials based on Bayes’ theorem, it rapidly converges on the best parameters for complex tasks like media or process optimization with minimal experimental cost.	To optimize the experimental formula and complex process parameters to greatly reduce the number of “trial and error” experiments.	1. Optimize the best concentration combination of various growth factors in MSC serum-free culture medium. 2. Optimize the 3D printing process parameters (e.g., printing speed and temperature) of biological materials to obtain the best molding accuracy.
	Reinforcement Learning (RL)	An agent learns optimal decision-making strategies through interaction with a dynamic environment, ideal for controlling multi-step, adaptive biological processes. This technique empowers bioprocesses to master complex, long-term operations through autonomous decision-making. It intelligently adjusts system parameters in real-time, enabling hands-off, adaptive control essential for sophisticated MSC manufacturing and tissue engineering.	To provide the decision-making framework for autonomous bioprocess control, allowing systems like bioreactors or differentiation protocols to self-optimize based on real-time feedback.	1. Control the dynamic environment of the bioreactor (pH, dissolved oxygen, perfusion rate) to maximize cell yield. 2. Develop a dynamic strategy for adding factors to MSCs during differentiation.

## Data Availability

Not applicable.

## Supplementary Materials

The following supporting information can be downloaded at: https://www.mdpi.com/article/10.3390/bioengineering12121302/s1, Literature Search Strategy.
