# Supplementary material for: Artificial Intelligence Driven Innovation: Advancing Mesenchymal Stem Cell Therapies and Intelligent Biomaterials for Regenerative Medicine
Source: Bioengineering (Basel). 2025 Nov 26;12(12):1302. doi: 10.3390/bioengineering12121302 (PMC12729526; doi:10.3390/bioengineering12121302)
Supplement: Supplementary file 1 [file bioengineering-12-01302-s001.zip › bioengineering-3916138-supplementary.pdf]

Literature Search Strategy

Review Title: Artificial Intelligence Driven Innovation: Advancing Mesenchymal Stem Cell Therapies and Intelligent Biomaterials for Regenerative Medicine

Aim: The objective of this literature search was to comprehensively identify peer-reviewed research articles and significant preprints at the intersection of mesenchymal stem cell (MSC) biology, artificial intelligence (AI)/machine learning (ML), and biomaterials for regenerative medicine applications.

Databases:
The primary sources for the identification of relevant studies were the PubMed/MEDLINE, Web of Science, and Scopus electronic databases. The primary sources for the identification of relevant studies were the PubMed/MEDLINE, Web of Science, and Scopus electronic databases. To ensure thorough coverage of the literature, manual searches of the reference lists of all included articles and relevant review papers were also performed to identify additional potentially eligible studies.

Search Timeframe:
The search was limited to studies published within the last 5 years (September 2020–September 2025). This focus on recent literature was implemented to highlight the most current and impactful findings, reflecting the modern state of AI applications for MSC and biomaterials.

Search Keywords
For PubMed
("Artificial Intelligence"[Mesh] OR "Machine Learning"[Mesh] OR "Deep Learning"[Mesh] OR "Neural Networks, Computer"[Mesh] OR "AI" OR "convolutional neural network*" OR CNN OR "generative adversarial network*" OR GAN OR "random forest" OR "support vector machine*" OR SVM OR "Bayesian optimization" OR "natural language processing" OR NLP)AND(("Mesenchymal Stem Cells"[Mesh] OR "Mesenchymal Stromal Cells"[Mesh] OR "Stromal Cells"[Mesh] OR MSC OR MSCs) OR ("Biocompatible Materials"[Mesh] OR "Biomimetic Materials"[Mesh] OR biomaterial* OR scaffold* OR hydrogel* OR "tissue engineering"[Mesh] OR "extracellular matrix mimics") AND ("Regenerative Medicine"[Mesh] OR "regenerative medicin*" OR "tissue regeneration"[Mesh] OR "bone regeneration"[Mesh] OR "cartilage regeneration" OR "wound healing"[Mesh]))

For Web of Science
(("Artificial Intelligence" OR "Machine Learning" OR "Deep Learning" OR "Neural Networks" OR "AI" OR "convolutional neural network*" OR CNN OR "generative adversarial network*" OR GAN OR "random forest" OR "support vector machine*" OR SVM OR "Bayesian optimization" OR "natural language processing" OR NLP))
AND(("Mesenchymal Stem Cells" OR "Mesenchymal Stromal Cells" OR "Stromal

Cells" OR MSC OR MSCs) OR ("Biocompatible Materials" OR "Biomimetic Materials" OR biomaterial* OR scaffold* OR hydrogel* OR "tissue engineering" OR "extracellular matrix mimics"))AND(("Regenerative Medicine" OR "regenerative medicin*" OR "tissue regeneration" OR "bone regeneration" OR "cartilage regeneration" OR "wound healing"))

For Scopus

(TITLE-ABS-KEY ("Artificial Intelligence" OR "Machine Learning" OR "Deep Learning" OR "Neural Networks" OR "AI" OR "convolutional neural network*" OR CNN OR "generative adversarial network*" OR GAN OR "random forest" OR "support vector machine*" OR SVM OR "Bayesian optimization" OR "natural language processing" OR NLP))AND(TITLE-ABS-KEY ("Mesenchymal Stem Cells" OR "Mesenchymal Stromal Cells" OR "Stromal Cells" OR MSC OR MSCs OR "Biocompatible Materials" OR "Biomimetic Materials" OR biomaterial* OR scaffold* OR hydrogel* OR "tissue engineering" OR "extracellular matrix mimics"))
AND(TITLE-ABS-KEY ("Regenerative Medicine" OR "regenerative medicin*" OR "tissue regeneration" OR "bone regeneration" OR "cartilage regeneration" OR "wound healing"))

Inclusion and Exclusion Criteria

For MSCs section

Inclusion Criteria:

1. Studies must apply a core AI/ML methodology (e.g., supervised/unsupervised/deep learning) for analysis, prediction, or design.
2. Studies must involve human or animal MSCs in vitro or in vivo.
3. Publication types: Primary research articles, review articles, and influential conference proceedings.
4. Language: English only.
5. Date Range: 2020–2025.

Exclusion Criteria:

1. Studies that do not primarily involve MSCs (e.g., focused on other stem cells like iPSCs or embryonic stem cells, or primary differentiated cells).
2. Studies where the computational method without a core AI/ML component.
3. Studies that only use AI for administrative purposes (e.g., patient record management) rather than for direct scientific discovery related to MSCs.
4. Editorials, letters, or non-peer-reviewed commentaries (except for the preprints specified above).
5. Studies for which the full text is not accessible.

For Biomaterials section

Inclusion Criteria:

1. Studies must apply a core AI/ML methodology (e.g., supervised/unsupervised/deep learning) for analysis, prediction, or design.

2. The study must involve the design, fabrication, functionalization, or testing of a defined biomaterial system.
3. Publication types: Primary research articles, review articles, and influential conference proceedings.
4. Language: English only.
5. Date Range: 2020–2025.

Exclusion Criteria:
1. Studies that do not primarily involve biomaterials.
2. Studies where the computational method without a core AI/ML component.
3. Studies that only use AI for administrative purposes (e.g., patient record management) rather than for direct scientific discovery related to biomaterials.
4. Editorials, letters, or non-peer-reviewed commentaries (except for the preprints specified above).
5. Studies for which the full text is not accessible.

Study Selection and Data Management
Search results from all databases were exported to reference management software EndNote for deduplication. The study selection process was conducted in two phases:
1. Title and Abstract Screening: The retrieved records were initially screened based on their titles and abstracts against the inclusion/exclusion criteria.
2. Full-Text Review: The full text of potentially relevant articles was then assessed for final inclusion.
The above screening process was conducted independently by two researcher (Mengyu HUANG, Ying Liu), with any discrepancies resolved through consensus.

Search Results
The initial systematic search across PubMed, Web of Science, and Scopus yielded a total of 1810 records. Supplemental manual searching of reference lists and relevant review articles identified an additional 154 records for potential inclusion. After removing 529 duplicates, 1453 unique articles remained for screening. The study selection was performed in two stages. First, the titles and abstracts of these records were screened against the inclusion and exclusion criteria, which excluded 1114 articles. Subsequently, the full texts of the remaining 321 articles were thoroughly assessed for eligibility. This process led to the exclusion of 50 additional studies, with reasons for exclusion documented. Ultimately, a total of 271 studies were deemed relevant and included for qualitative synthesis in this review. These studies form the foundational references for this review.

Review writing

Due to space constraints, although our search strategy identified a substantial number of publications, not all included studies are cited in this review. The study selection

criteria were based on methodological rigor, innovativeness and its contribution to domain knowledge, as well as relevance to the core arguments. For multiple highly similar studies on analogous topics published by the same research team, only the most recent and groundbreaking findings were selected.
